# Audiological and Speech Performance After Cochlear Implantation in Cochlear Aplasia Deformity

**DOI:** 10.7759/cureus.16654

**Published:** 2021-07-27

**Authors:** Salman F Alhabib

**Affiliations:** 1 College of Medicine, King Abdullah Ear Specialist Center, King Saud University, Riyadh, SAU

**Keywords:** inner ear malformation, cochlear aplasia, common cavity, cochlear implant, outcomes

## Abstract

Inner ear malformation is a congenital anomaly associated with prelingual sensory neural hearing loss in the pediatric population. This is a case report of bilateral radiologically diagnosed cochlear aplasia in a child who underwent unilateral left cochlear implantation with audiological results at one-year follow-up after surgery. Sixteen months after the cochlear implantation surgery, the child could produce 200 words and say a sentence with two words. In certain cases of inner ear malformation, the subgrouping of cystic cavity can be difficult and should not delay the trial of cochlear implant provided an acceptable anatomical appearance of the inner ear with cochleovestibular nerve existence and a proper electrode used for implantation.

## Introduction

Common cavity (CC) deformity is one of the congenital inner ear malformations (IEMs) associated with profound sensorineural hearing loss (SNHL) in children. It is defined as a cystic cavity in the inner ear without showing any structural differentiation between the cochlea and vestibule [1]. CC is the second most common congenital IEM accounting 25% of profound SNHL related to IEM and it is caused by the arrest of the differentiation of the otocyst into the cochlea and vestibule during the fourth gestational week [2]. In some cases of IEM, accurate identification of CC and differentiation from cochlear aplasia (CA) on radiological images is important as the latter is considered a contraindication for cochlear implantation (CI) [3]. CA is defined as the absence of the cochlea with a variable size of the vestibule and all or parts of the semicircular canals [1,4,5]. These definitions are based on the structural appearance of the inner ear and its position relative to the internal auditory canal (IAC) on computed tomography (CT) of the temporal bone. If the cavity is extended anteriorly and posteriorly to the IAC, the condition is called CC, and if the cavity is located only posteriorly to the internal auditory meatus (IAM), the term CA is used [1,4,5]. CI was considered contraindicated for both CC and CA, and related to the existence of cochlear neural structures, stability of implanted electrodes inside the cavity, structural availability of the cochlear nerve, and the risk of cerebrospinal fluid gusher and meningitis [6,7]. Several studies have found that CI in patients with CC is effective and has hearing and speech benefits [7,8]. However, the audiological outcomes of CI in patients with CC were found to be not good as the other IEM [9]. The aim of this study is to present the management for a case of bilateral IEM associated with prelingual profound SNHL with radiological diagnosis of CA represented bilaterally.

## Case presentation

Here, we present a case of bilateral congenital IEM. A four-year-old female patient who was diagnosed with congenital profound SNHL at the age of 18 months presented to our otology clinic for evaluation and advice. No medical problems in the mother were recorded during pregnancy. The child was delivered at full term via a caesarian section. The mother and baby were not admitted to the intensive care unit. The normal pediatric examination was performed, and the child was sent home three days after delivery.

Hearing screening test was not performed after delivery. Hearing loss was observed by the parents at six months of age. Apart from the hearing loss, the child had normal growth and development with a normal facial appearance. Microscopic examination revealed normal bilateral tympanic membrane.

**Figure 1:**
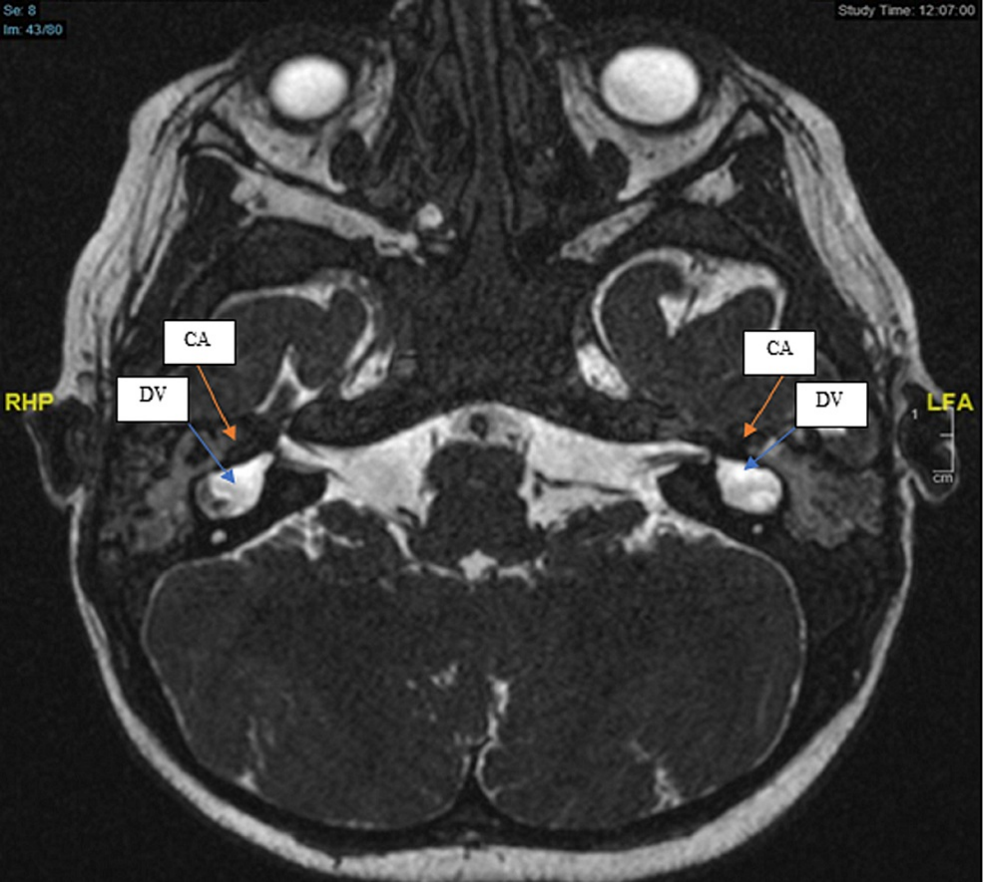
MRI internal auditory canal showed bilateral cochlear aplasia (CA), the dilated vestibule (DV).

**Figure 2:**
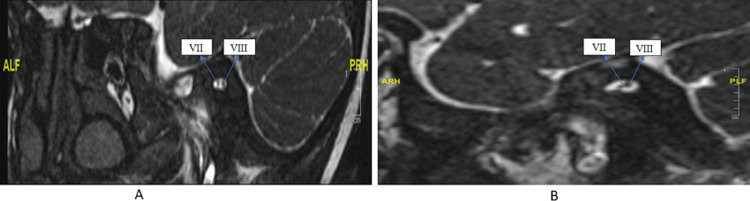
(A) MRI internal auditory canal showed right IAC has two nerves that may represent both the facial nerve (VII) and cochleovestibular nerve (VIII), and (B) MRI internal auditory canal showed left IAC has two nerves that may represent both the facial nerve (VII) and cochleovestibular nerve (VIII). IAC: Internal auditory canal

The otoacoustic emissions test was performed at 12 months of age, and no emissions were detected. The auditory brainstem response (ABR) was assessed at the age of 12 months, and no reproducible waves at 90 dB presentation level were observed. Hearing aids for both ears were used for three months with no benefit. Furthermore, magnetic resonance imaging (MRI) of the temporal bone revealed a bilateral CA with a dilated vestibule and a malformed semicircular canal located posteroinferiorly to the IAC, both of which contain two nerves that may represent both the facial and cochleovestibular nerves with larger sizes in the left ear (Figures 1-2 show the axial and the parasagittal view of both sides, respectively). According to the IAC grading system, grade II represents the presence of nerves in the parasagittal plane on both sides [10].

Treatment plan

**Figure 3:**
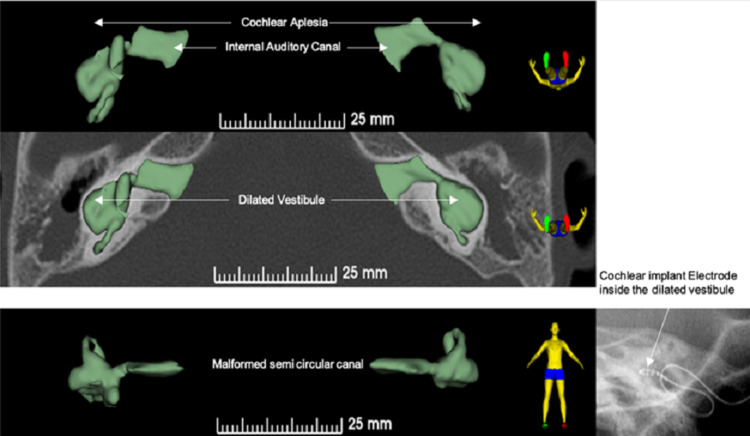
The preoperative planning using a 3D reconstruction for the CT findings of the studied case.

**Figure 4:**
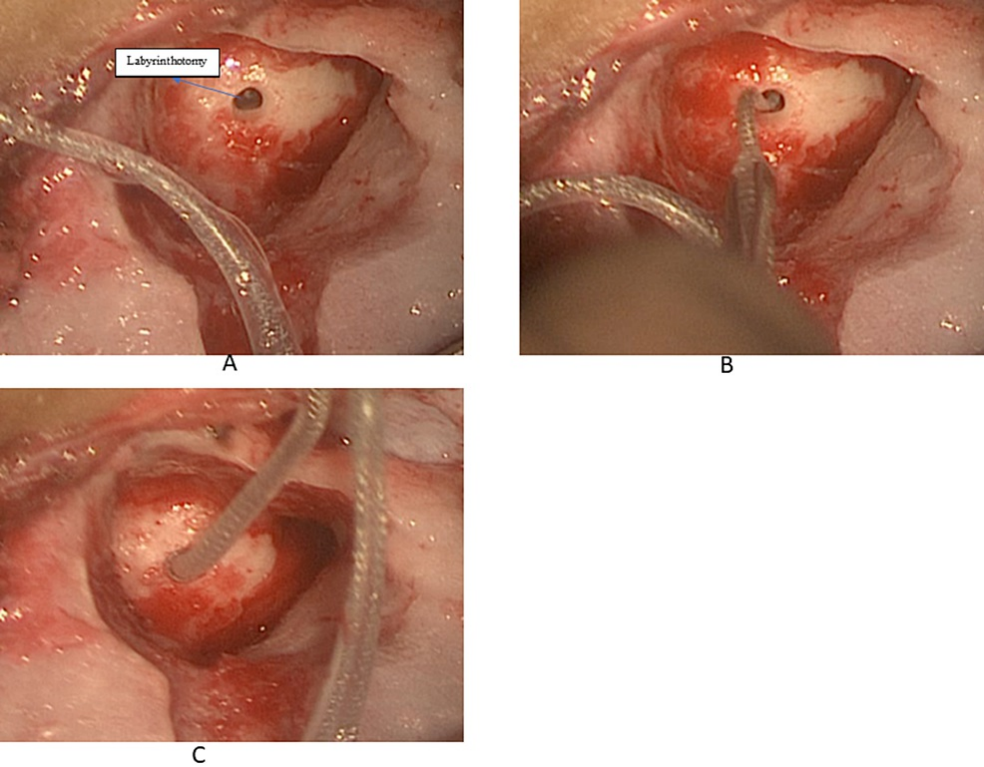
An illustration for the trans-mastoid labyrinthotomy approach showing (a) single slit TML, (b) insertion of the Form19 electrodes, and (c) full insertion of the electrode with the complete closure of the labyrinthotomy with silicon ring (cork). TML: Trans-mastoid labyrinthectomy

Based on the clinical history and audiological and 3D reconstruction of the radiological findings (Figure 3), the CI committee recommended unilateral CI for the left ear due to bigger cystic cavity and thicker eighth cranial nerve before considering an auditory brain stem implantation. After the parents provided signed informed consent, the child received a CI at the age of 2.7 years. Form19 electrodes from MED-EL (Innsbruck, Austria) were used for the CI procedure. A single-slit trans-mastoid labyrinthectomy (TML) approach was used with a smooth insertion of the electrode. No oozing or gushers were observed during the TML approach. Figure 4 shows the approach, insertion technique, and closure of the labyrinthectomy opening.

The intraoperative objective measurements showed an electrically evoked compound action potential (ECAP) in two of the 12 electrodes (3 and 7) with normal impedance values for all electrodes. Intraoperative mastoid radiation (X-ray) showed complete insertion of the electrode with an electrode located in the lateral wall and no penetration inside the IAC. Normal wound healing with no adverse events occurred after the CI surgery.

Audiological and speech assessment outcomes

**Figure 5:**
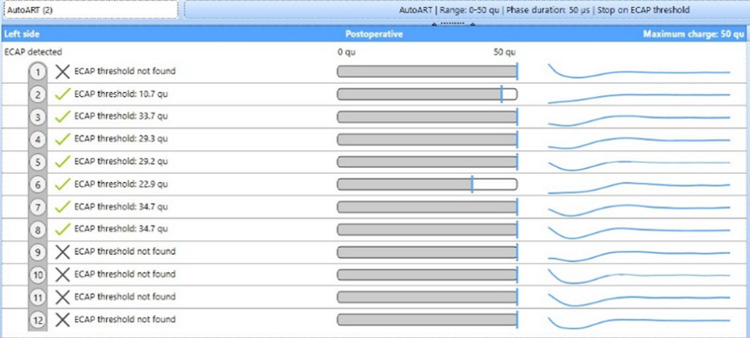
Eight-month post-operative evoked compound action potential (ECAP) showing responses in 7 electrodes.

**Figure 6:**
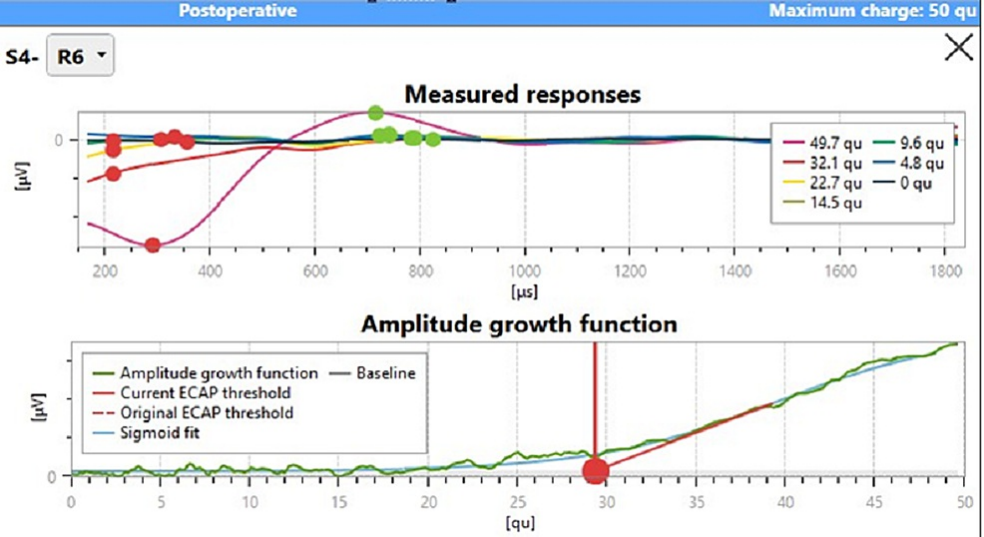
Example for the measured response and amplitude growth function of evoked compound action potential (ECAP) from electrode number 6.

The activation of the audio processor was performed three days after the CI surgery. Early activation of CI audioprocessor showed to be safe approach and more economic compared to classical activation that performed one month after the surgery [11]*.* Impedance field telemetry (IFT) was normal, and the ECAP was present only at electrodes 3 and 7. Four progressive maps were obtained. The child started to hear loud noise one month after the activation of the device. Based on the data logged, the average number of hours of wearing the audio processor was 12 h per day. Six months after the surgery, the pure tone average of the child was 32.5 dB, the speech reception threshold (SRT) was 20 dB, and the child tried to initiate some words, but the ECAPS was present in 7 of 12 electrodes (Figures 5, 6).

Eight months after the CI surgery, the child produced 16 words, and the language age judge was 20 months. Sixteen months after the CI surgery, the child could produce 200 words and say a sentence with two words. LittlEars Questionnaire was found to be valid and reliable screening tools for auditory skills in infants and toddlers who received cochlear implants [12]. The responses of the parents to the LittlEars Questionnaire, when the child was 4 years old, showed a score of 32, indicating normal hearing for the corresponding age.

## Discussion

Several studies subgroup the inner ear single cavities into three types according to their radiological appearance in the CT of the temporal bone [1,12,13]. First type is Michel’s otocyst deformity, characterized by a single cyst and complete absence of the IAC and cochlear nerve. Second type is the CA deformity has a vestibule with variable shape with all or portions of the semicircular canals but with complete absence of the cochlea based on the entrance of the IAC, which is located anteromedially to the cavity, and with absence of cochlear nerve. Third type is the CC deformity, which has a single cavity with all or part of the semicircular canal, and the IAC presents with an identifiable cochlear nerve entering at the middle of the cavity [1].

According to IEM classification, our patient had a radiological diagnosis of CA with a grade II IAC classification on MRI on both sides. However, this patient showed remarkable language development after CI surgery in the left ear. Additionally, this result strongly motivated the parents and the child to ask for implantation in the contralateral ear eight months after the first CI surgery. The LittlEars Questionnaire was administered to the parents when the child was 4 years old, and the responses showed an acceptable hearing level corresponding to her age. Jeong and Kim presented two cases of CI in a radiological diagnosis of CA that showed good speech perception abilities in open set monosyllabic word tests [14]. The differentiation of CC from CA in certain cases is difficult, and using an IEM classification based on radiological appearance should not delay a trial of CI if no anatomical barrier, such as the absence of the IAM, cochlear nerve, or hypoplastic cyst, contraindicates the surgery. Classification of Jackler and Sennaroglu is widely accepted but few malformations are intervening between the typical forms [1,2]. Therefore, the final diagnosis to our patient based on the auditory development is a CC deformity located posteriolateral to the IAC.

Several studies have shown audiological and speech benefits after CI surgery for IEM deformities, but these benefits are found to be inferior to those of CI surgery in patients with normal inner ears [8,9,13]. In general, several factors, such as age at implantation, cognitive ability, additional disability, residual hearing, parental participation, and the daily use of the cochlear implant audio processor, affect language development after CI [14].In addition, other factor that could affect the language development in CC patients is the type of electrode used for implantation as the modiolus hugging electrode is not advised in this deformity as it stays in the middle of the cavity without direct contact to the neural tissues that are located on the lateral wall of the cavity [13,15].

Therefore, a lateral wall electrode is recommended for this type of deformity. Several surgical approaches have been described in the literature, including single-slit TML, double-slit TML, and the transmastoid with facial recess approaches. The latter is associated with a high incidence of facial nerve paralysis of up to 52%, which may be attributed to the abnormal facial nerve course in the temporal bone. The single slit TML is found to be easy, less time-consuming, less risky over the facial nerve and it provides easier control of the labyrinthotomy in the presence of a gusher compared to transfacial approach [13].

A Form 19 electrode from MED-EL was chosen as the electrodes array is laterally positioned and the electrodes are represented in both sides of the electrode array, so wider range of contact with the neural tissue. Furthermore, the electrode array has a ‘cork’ feature at the level of the silicon ring located at the end of insertion which proposes to efficiently seal the cochleostomy opening, therefore decreasing the incidence CSF leak after the CI surgery.

## Conclusions

In some cases, subgrouping of the cystic cavities for IEMs can be difficult and should not delay the trial of a cochlear implant provided that the anatomical appearance of the inner ear with cochleovestibular nerve existence is acceptable and an appropriate electrode is used for implantation. This case report demonstrates the audiological and speech benefits of cochlear implants in radiologically diagnosed CA.
